# Imaging patterns of hemorrhagic transformation after tenecteplase versus alteplase in real-world practice

**DOI:** 10.3389/fneur.2026.1823868

**Published:** 2026-07-17

**Authors:** Shijie Guo, Dong Zhang, Ying Zong, Dongrui Huang, Lingyun Liu, Yunhua Yue

**Affiliations:** Department of Neurology, Yangpu Hospital, School of Medicine, Tongji University, Shanghai, China

**Keywords:** alteplase, hemorrhagic transformation, imaging patterns, real-world practice, tenecteplase

## Abstract

**Background:**

While randomized trials have demonstrated noninferiority of tenecteplase compared with alteplase in terms of functional outcomes and symptomatic intracranial hemorrhage (sICH), differences in hemorrhagic imaging phenotypes remain insufficiently explored in real-world practice.

**Methods:**

We conducted a retrospective real-world cohort study of consecutive patients with acute ischemic stroke treated with intravenous thrombolysis between January and December 2025. The primary endpoint was ordinal shift in hemorrhagic transformation severity according to the Heidelberg Bleeding Classification (HBC). Secondary endpoints included sICH. Multivariable logistic regression was performed to identify independent predictors of hemorrhage.

**Results:**

A total of 95 patients were included (alteplase *n* = 41; tenecteplase *n* = 54). Baseline characteristics were largely comparable between groups. No significant difference in sICH incidence was observed. Although remote hemorrhagic phenotypes were numerically more frequent in the tenecteplase group, no significant ordinal shift was detected between treatment groups. In multivariable analysis, higher baseline NIHSS score (OR 1.10, 95% CI 1.02–1.18, *p* = 0.013) and longer ONT (OR 1.12 per 10 min, 95% CI 1.02–1.23, *p* = 0.016) were independently associated with intracranial hemorrhage, whereas thrombolytic agent type was not.

**Conclusion:**

In this small exploratory real-world cohort, tenecteplase was not associated with an obvious increase in hemorrhagic events compared with alteplase. However, because of the limited sample size and small number of severe hemorrhagic events, these findings should be interpreted as preliminary and hypothesis-generating. Imaging-based classification using the Heidelberg framework did not reveal a significant shift toward more severe hemorrhagic phenotypes.

## Introduction

Intravenous thrombolysis remains a cornerstone of acute ischemic stroke treatment (1, 2). For decades, alteplase has been the standard thrombolytic agent; however, tenecteplase has increasingly been adopted as an alternative because of its higher fibrin specificity, longer half-life, and single-bolus administration (3). Randomized trials and meta-analyses have demonstrated comparable efficacy between tenecteplase and alteplase in terms of early reperfusion and functional outcomes, symptomatic intracranial hemorrhage (sICH) (3–7). This has led to the growing use lead to the growing use of tenecteplase in routine clinical practice (8).

Hemorrhagic transformation remains the most feared complication of intravenous thrombolysis and a major determinant of early neurological deterioration and poor outcome (9–11). However, safety assessment in most thrombolysis trials has relied primarily on binary definitions of sICH (12). Hemorrhagic transformation (HT), in contrast, represents a biologically heterogeneous spectrum ranging from petechial hemorrhage to space-occupying parenchymal hematoma and remote bleeding (9). These imaging phenotypes may reflect distinct pathophysiological mechanisms including reperfusion injury, blood–brain barrier disruption, and systemic vascular vulnerability (13, 14).

Whether tenecteplase and alteplase differ in hemorrhagic imaging phenotypes in real-world practice remains insufficiently explored (15, 16). Beyond absolute hemorrhage rates, understanding severity distribution and spatial patterns may provide mechanistic insight and inform risk stratification (9, 17).

Therefore, we conducted a real-world observational study to compare hemorrhagic transformation severity and imaging phenotypes following intravenous tenecteplase versus alteplase using the Heidelberg Bleeding Classification (HBC), with ordinal shift in hemorrhagic severity as the primary endpoint (18).

## Methods

### Study design and population

We conducted a retrospective, real-world observational cohort study of consecutive patients with acute ischemic stroke treated with intravenous thrombolysis at Yangpu District Central Hospital of Shanghai (Yangpu Hospital Affiliated to Tongji University) stroke center between January 2025 and December 2025. Patients received either intravenous alteplase (0.9 mg/kg, maximum 90 mg; 10% bolus followed by 60-min infusion) or intravenous tenecteplase (0.25 mg/kg, maximum 25 mg; single bolus) according to institutional protocols and drug availability during the study period.

Definitions of vascular risk factors were based on medical records and laboratory data during hospitalization. Smoking was defined as current or previous smoking documented in the medical record. Drinking was defined as regular alcohol consumption documented in the medical record. Diabetes mellitus was defined as a previous diagnosis of diabetes, current use of glucose-lowering medication, or blood glucose/HbA1c levels meeting diagnostic criteria during hospitalization. Hyperlipidemia was defined as a previous diagnosis, current use of lipid-lowering medication, or an abnormal lipid profile during hospitalization.

Eligible patients met the following criteria:

(1) Age ≥ 18 years;
(2) Clinically diagnosed with acute ischemic stroke based on symptoms, neurological examination, and baseline neuroimaging;
(3) Treatment with intravenous alteplase or tenecteplase within guideline-recommended time windows;
(4) Availability of baseline brain imaging and follow-up imaging within 24 h after thrombolysis.

Patients were excluded if they:

(1) Underwent primary mechanical thrombectomy without prior intravenous thrombolysis;
(2) Lacked imaging;
(3) Had pre-existing intracranial hemorrhage on baseline imaging.
(4) Had contraindications to intravenous thrombolysis, including pregnancy, severe hepatic or renal dysfunction, coagulation abnormalities, or intracranial tumors.

The study was approved by the institutional review board, and the requirement for written informed consent was waived because of the retrospective design.

### Outcomes

The primary endpoint was ordinal shift in hemorrhagic transformation severity according to Heidelberg Bleeding Classification (HBC).

The secondary endpoint was symptomatic intracranial hemorrhage, defined according to the SITS-MOST and ECASS-3 criteria.

## Statistical analysis

**Figure 1:**
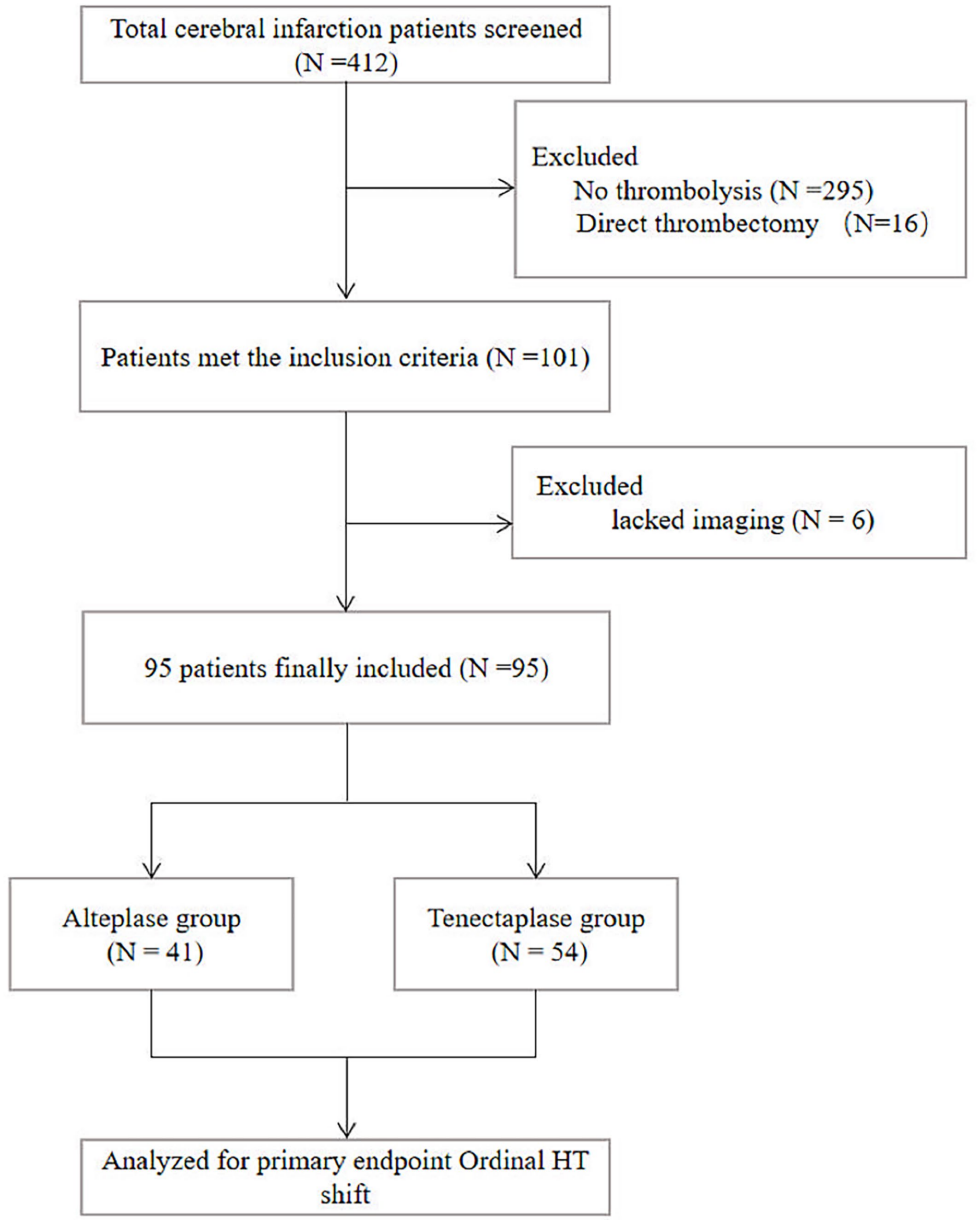
Flowchart of patient selection.

**Figure 2:**
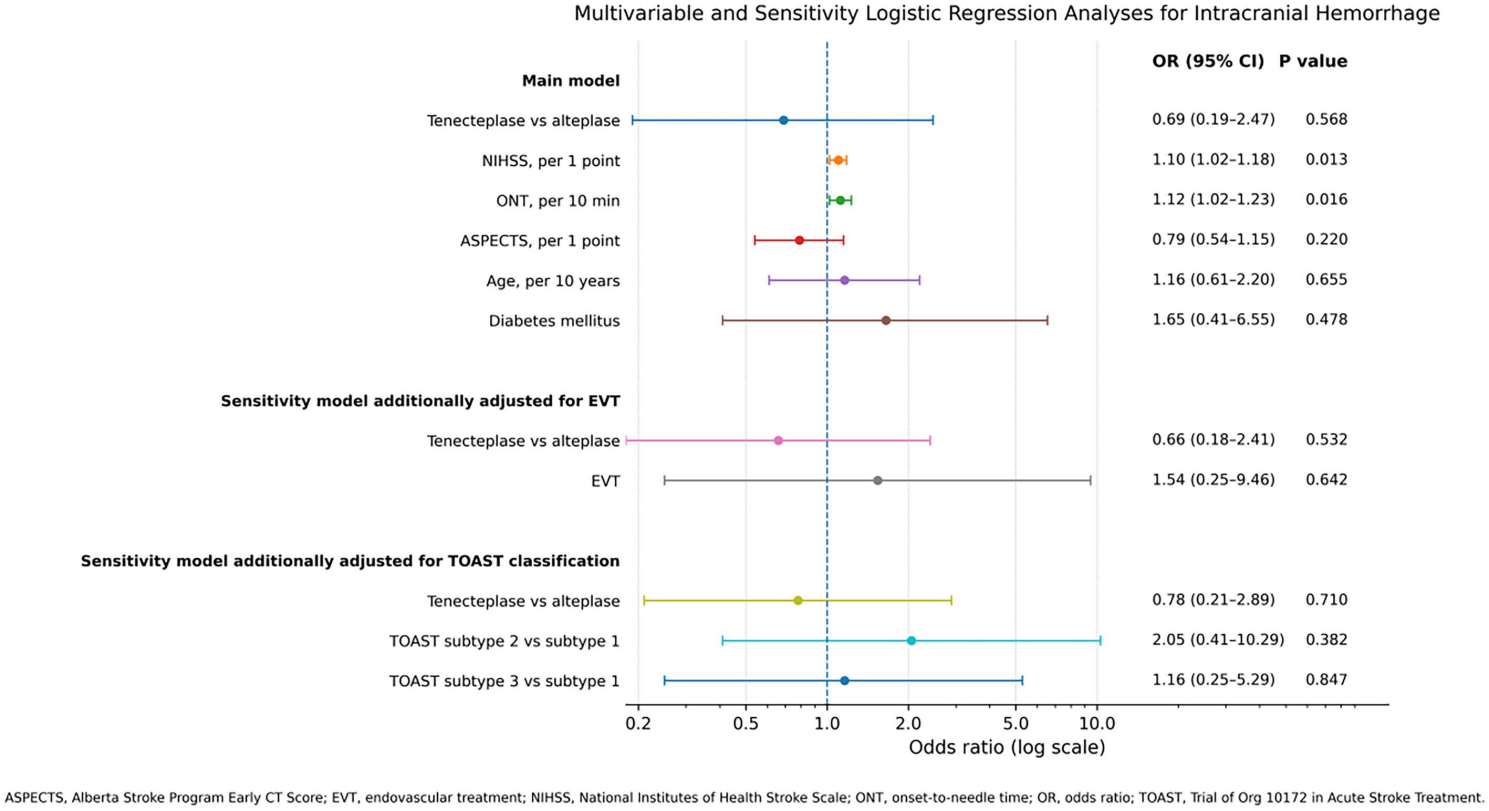
Forest plot of multivariate logistic regression analysis for sICH.

**Figure 3:**
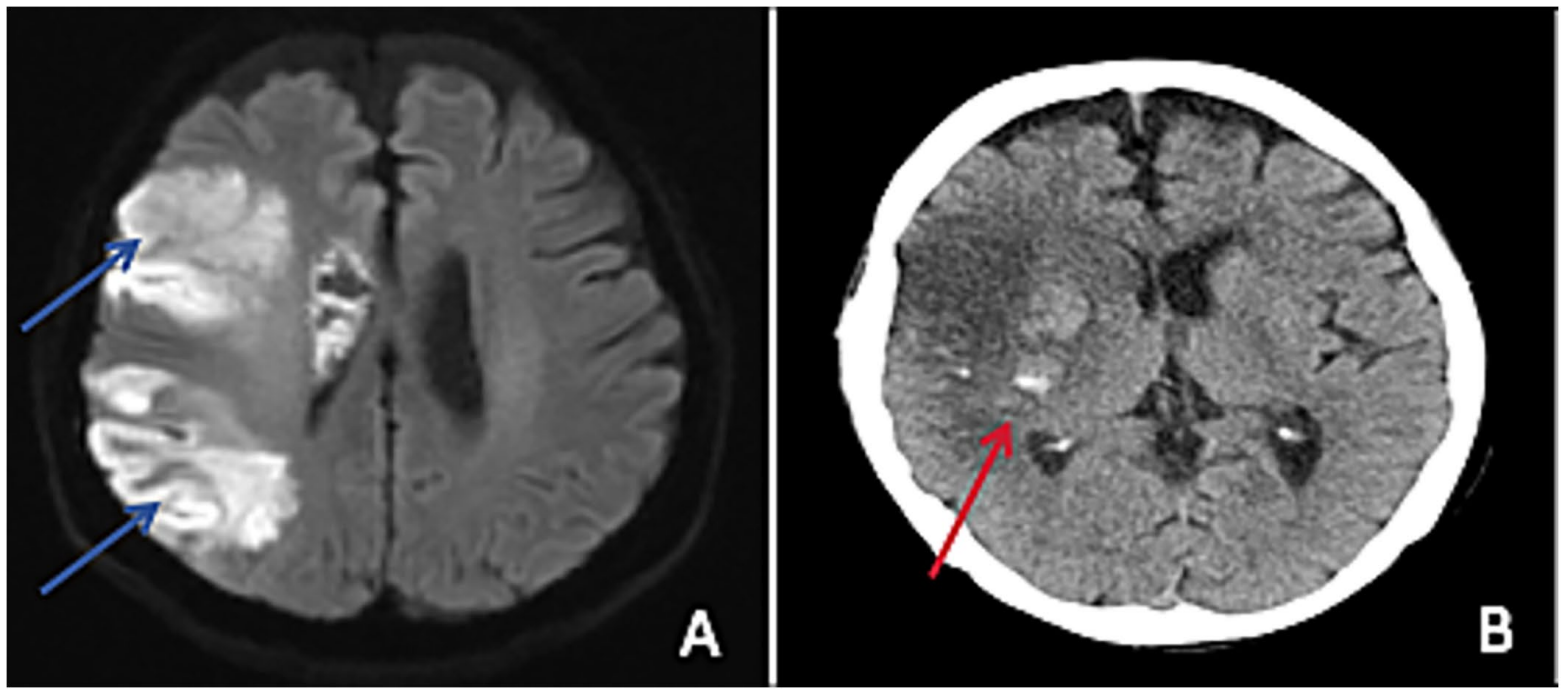
Representative case of HBC-PH hemorrhagic transformation following intravenous alteplase thrombolysis. **(A)** Diffusion-weighted magnetic resonance imaging (DWI) demonstrates restricted diffusion consistent with acute cerebral infarction (blue arrows). **(B)** Non-contrast head computed tomography (CT) shows a hyperdense intraparenchymal hemorrhage corresponding to hemorrhagic transformation (red arrows).

**Figure 4:**
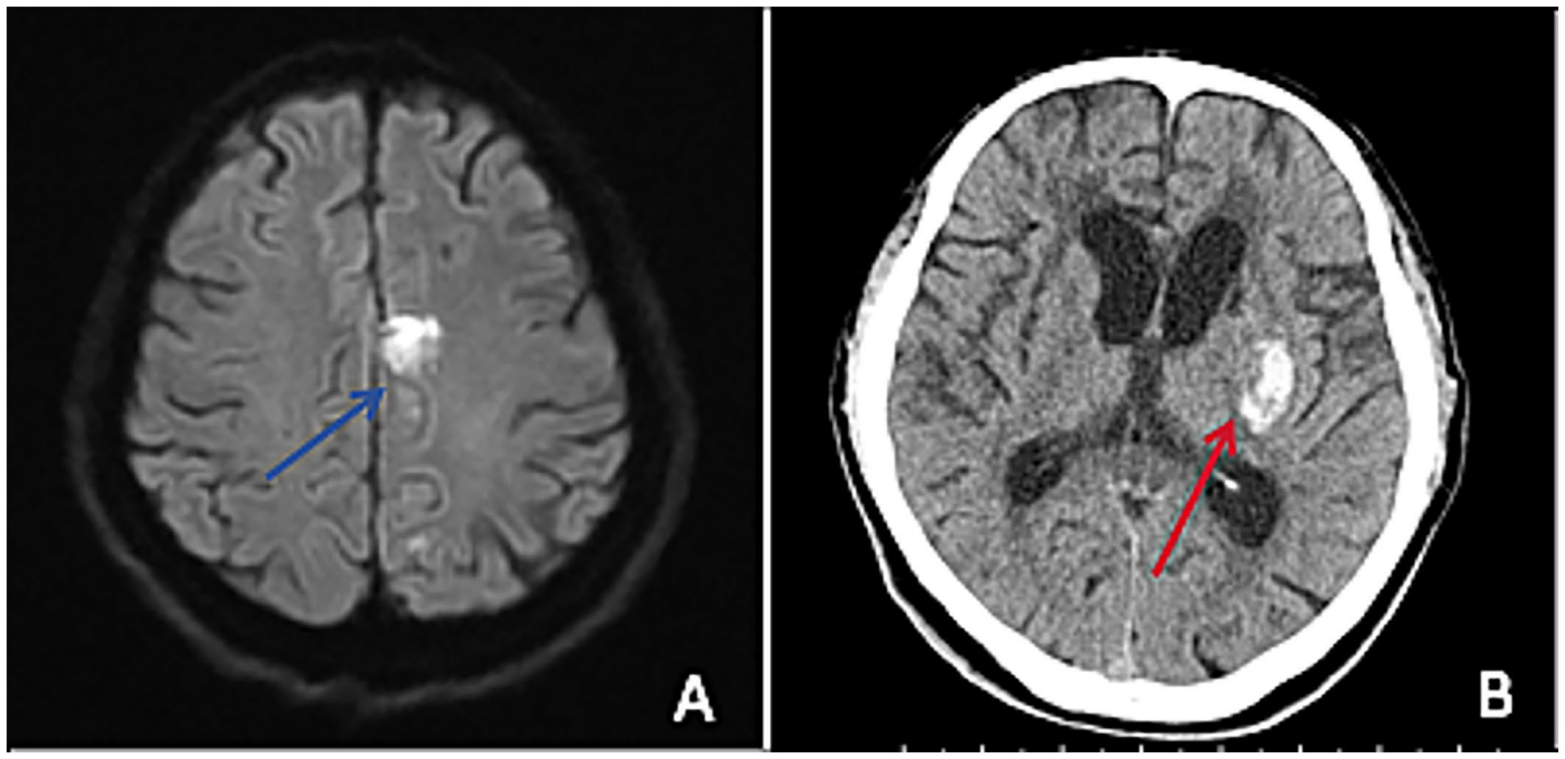
A case of HBC-3a hemorrhagic transformation following intravenous Tenecteplase thrombolysis. **(A)** Diffusion-weighted magnetic resonance imaging (DWI) demonstrates restricted diffusion consistent with acute cerebral infarction (blue arrows). **(B)** Non-contrast head computed tomography (CT) shows a hyperdense intraparenchymal hemorrhage corresponding to hemorrhagic transformation (red arrows).

**Figure 5:**
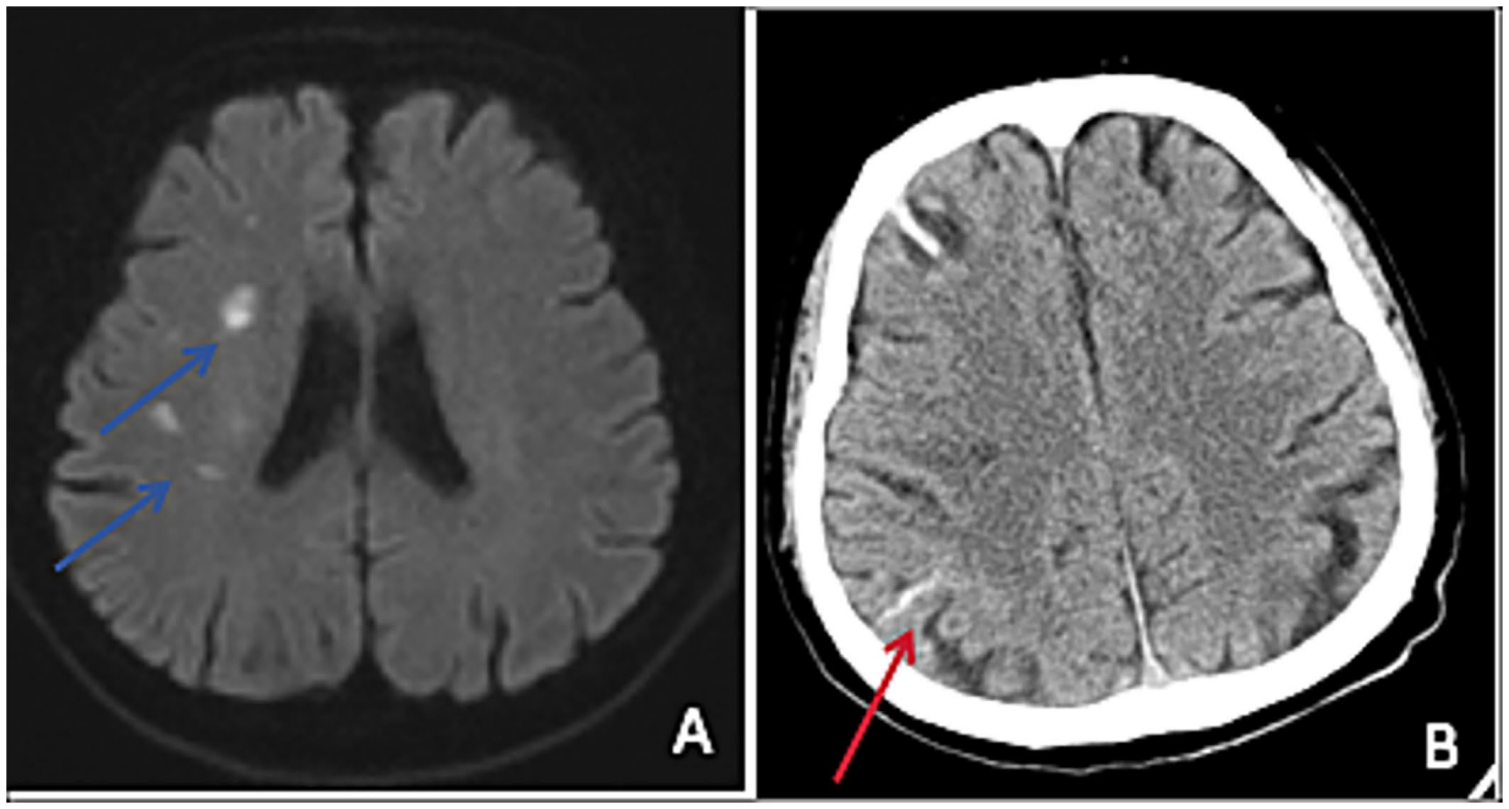
A case of HBC-3c hemorrhagic transformation following intravenous tenecteplase thrombolysis. **(A)** Diffusion-weighted magnetic resonance imaging (DWI) demonstrates restricted diffusion consistent with acute cerebral infarction (blue arrows). **(B)** Non-contrast head computed tomography (CT) shows hyperdense subarachnoid hemorrhage corresponding to hemorrhagic transformation (red arrows).

**Figure 6:**
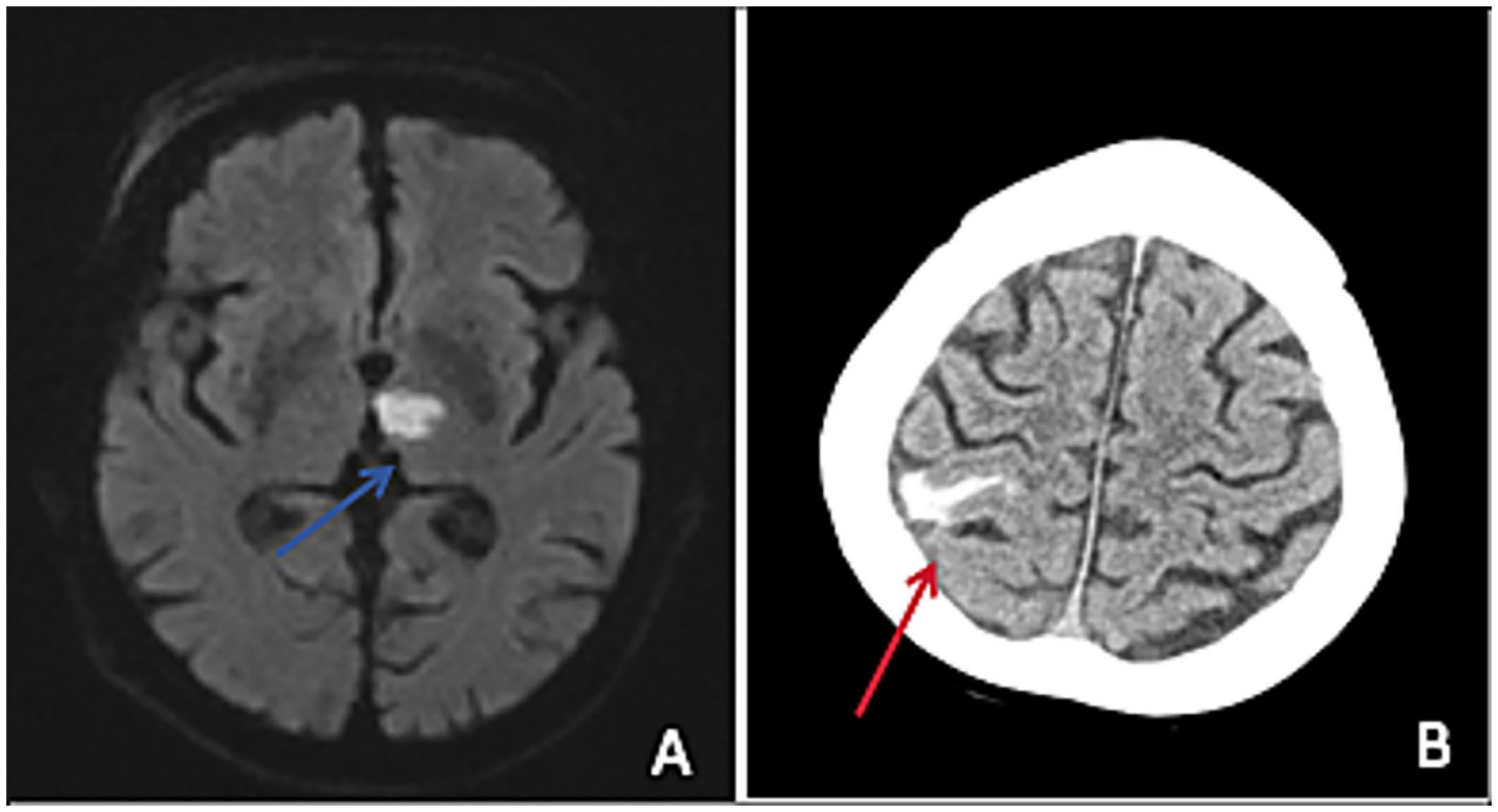
A case of HBC-3c hemorrhagic transformation following intravenous tenecteplase thrombolysis. **(A)** Diffusion-weighted magnetic resonance imaging (DWI) demonstrates restricted diffusion consistent with acute cerebral infarction (blue arrows). **(B)** Non-contrast head computed tomography (CT) shows a subarachnoid hemorrhage corresponding to hemorrhagic transformation (red arrows).

Statistical analyses were performed using SPSS software. Continuous variables were compared using *t*-tests or Mann–Whitney U tests, while categorical variables were analyzed with *χ*^2^ tests or Fisher’s exact test. Baseline characteristics and outcome measures were compared across different medication groups. The presence or absence of intracerebral hemorrhage (sICH) was used as the dependent variable, and a multiple logistic regression model was employed for analysis. A prespecified parsimonious forced-entry logistic regression model was used, including treatment group, baseline NIHSS score, onset-to-needle time, ASPECTS, and age (Figures 1–6).

## Results

Among 95 patients (alteplase *n* = 41; tenecteplase *n* = 54), Baseline characteristics were generally comparable between groups. There were no significant differences in age, sex, baseline NIHSS score, blood pressure, atrial fibrillation, wake-up stroke, fall history, mechanical thrombectomy, ASPECTS, large-/medium-vessel involvement, or time from thrombolysis to follow-up imaging. Diabetes mellitus was more frequent in the tenecteplase group than in the alteplase group (35.19% vs. 14.63%, *p* = 0.024), Baseline ASPECTS and the proportion of patients with baseline vessel involvement/occlusion/stenosis were not significantly different between groups (Table 1).

**Table 1:** Demographic and clinical characteristics of the patients.

Variables	Alteplase (*N* = 41)	Tenecteplase (*N* = 54)	*p*-value
Age (years), median (IQR)	74 (68, 82)	70 (66, 81)	0.468
Male, *n* (%)	17 (41.46)	19 (35.19)	0.532
Risk factors, *n* (%)			
Smoking	15 (36.59)	21 (38.89)	0.819
Drinking	7 (17.07)	10 (18.52)	0.856
Diabetes	6 (14.63)	19 (35.19)	0.024
Prior ischemic stroke	6 (14.63)	15 (27.78)	0.112
Prior TIA	1 (2.44)	1 (1.85)	1.000
Hypertension	29 (70.73)	41 (75.93)	0.569
Hyperlipidemia	3 (7.32)	3 (5.56)	1.000
Coronary artery disease	5 (12.20)	10 (18.52)	0.403
Atrial fibrillation	11 (26.83)	8 (14.81)	0.147
Clinical data			
Wake-up stroke, *n* (%)	7 (17.07)	4 (7.41)	0.257
Fall, *n* (%)	2 (4.88)	2 (3.70)	1.000
mRS 0–2 on admission, *n* (%)	39 (95.12)	53 (98.15)	0.808
NIHSS on admission, median (IQR)	6 (3, 10)	4 (2, 9)	0.136
Systolic blood pressure (mmHg), median (IQR)	151.00 (141.00, 162.00)	152.50 (138.50, 167.00)	0.982
Diastolic blood pressure (mmHg), median (IQR)	86.00 (77.00, 94.00)	82.00 (73.50, 88.00)	0.167
Time from onset-to-needle (min), median (IQR)	116.00 (85.00, 187.00)	119.00 (94.00, 166.75)	0.550
Mechanical thrombectomy, *n* (%)	6 (14.63)	10 (18.52)	0.616
TOAST classification, *n* (%)			
Large-artery atherosclerosis	13 (31.71)	26 (48.15)	0.055
Cardioembolic	11 (26.83)	5 (9.26)	
Small-vessel occlusion	17 (41.46)	23 (42.59)	
ASPECTS, median (IQR)	8 (7–9)	9 (8–9)	0.338
Large-or-medium vessel involvement	7 (17.07)	10 (18.52)	0.856
Time from thrombolysis to follow-up imaging, median (IQR), h	24.0 (20.0–24.0)	24.0 (20.8–24.0)	0.484

In the main model, higher NIHSS (OR 1.10, 95% CI 1.02–1.18, *p* = 0.013) and longer ONT (OR 1.12 per 10 min, 95% CI 1.02–1.23, *p* = 0.016) were associated with hemorrhage. Treatment group was not independently associated with hemorrhage (tenecteplase vs. alteplase: OR 0.69, 95% CI 0.19–2.47, *p* = 0.568). The treatment-group estimate remained non-significant after separately adding EVT (OR OR 0.66, 95% CI 0.18–2.41, *p* = 0.532) and TOAST classification (OR 0.78, 95% CI 0.21–2.89, *p* = 0.710).

When hemorrhagic transformation severity was categorized into no hemorrhage, HI, and severe hemorrhage (PH2/remote/SAH), the distribution did not demonstrate a statistically significant ordinal shift between treatment groups. Although severe hemorrhage was numerically more frequent in the tenecteplase group, no statistically significant ordinal shift was detected; The small number of high-grade and remote hemorrhagic events limits the precision of this comparison (Tables 2–5).

**Table 2:** Multivariable logistic regression analyses for symptomatic intracranial hemorrhage.

Variable	OR (95% CI)	*p* value
Tenecteplase vs. alteplase	0.69 (0.19–2.47)	0.568
NIHSS	1.10 (1.02–1.18)	0.013
Time from onset-to-needle	1.12 (1.02–1.23)	0.016
ASPECTS	0.79 (0.54–1.15)	0.220
Age	1.16 (0.61–2.20)	0.655
Diabetes	1.65 (0.41–6.55)	0.478

The main parsimonious model included treatment group, NIHSS, ONT, ASPECTS, Diabetes and age. Sensitivity models separately added EVT and TOAST classification. ONT was scaled per 10 min and age per 10 years. NIHSS, National Institutes of Health Stroke Scale.

**Table 3:** Sensitivity logistic regression analyses for symptomatic intracranial hemorrhage.

Sensitivity model	Variable	OR (95% CI)	*p* value
Additionally adjusted for EVT	Tenecteplase vs. alteplase	0.66 (0.18–2.41)	0.532
	EVT	1.54 (0.25–9.46)	0.642
Additionally adjusted for TOAST classification	Tenecteplase vs. alteplase	0.78 (0.21–2.89)	0.710
	TOAST subtype 2 vs. subtype 1	2.05 (0.41–10.29)	0.382
	TOAST subtype 3 vs. subtype 1	1.16 (0.25–5.29)	0.847

The main parsimonious model included treatment group, NIHSS, ONT, ASPECTS, and age. Sensitivity models separately added EVT and TOAST classification.

**Table 4:** HBC in sICH.

HBC	Alteplase (*n* = 41)	Tenecteplase (*n* = 54)
1	0	0
1a	0	0
1b	1	1
1c	3	2
2 Ph2	1	0
3	0	0
3a PH remote	0	2
3b IVH	0	0
3c SAH	1	4
3d SDH	0	0

HBC, Heidelberg Bleeding Classification; sICH, symptomatic intracranial hemorrhage; HI1, hemorrhagic infarctiontype1; HI2, hemorrhagicinfarctiontype2; PH1, parenchymalhematomatype1; PH2, parenchymalhematomatype2; IVH, intraventricularhemorrhage; SAH, subarachnoidhemorrhage; SDH, subduralhematoma.

**Table 5:** Remote hemorrhagic characteristics.

ID	IVT agent	HBC	Age	HTN	DM	AF	Prior stroke	Fall	Affected circulation	NIHSS (arrival)	Thrombectomy	ONT (min)	DNT (min)	TOAST	Dimer (mg/L)	Glucose (mmol/L)	Platelets (×10^9^/L)
1	Tenecteplase	3c	68	1	1	0	1	0	Anterior	1	0	159	78	1	0.22	6.0	131
2	Alteplase	PH2	69	0	0	0	0	0	Anterior	1	0	237	25	3	1.21	6.79	191
3	Tenecteplase	3c	58	0	0	0	0	0	Anterior	4	0	50	31	3	0.29	4.27	418
4	Tenecteplase	3c	93	0	0	0	1	0	Posterior	13	0	223	43	3	6.88	12.34	186
5	Tenecteplase	3c	90	1	1	0	1	1	Posterior	12	0	78	23	1	2.66	7.58	213
6	Alteplase	3c	91	1	0	1	0	1	Anterior	25	1	30	21	1	7.39	17.73	119
7	Tenecteplase	3a	70	1	0	0	0	0	Anterior	29	1	194	46	1	2.31	4.63	609
8	Tenecteplase	3a	77	1	0	0	0	0	Anterior	6	1	140	30	1	1.48	9.99	252

HBC, Heidelberg Bleeding Classification; DNT, Door-to-Needle Time; ONT, Onset-to-Needle Time; HNT, Hypertension.

## Discussion

In this real-world small exploratory cohort of patients with acute ischemic stroke treated with intravenous thrombolysis, we identified three principal findings. First, the overall incidence of symptomatic intracranial hemorrhage (sICH) did not differ significantly between tenecteplase and alteplase, consistent with prior randomized trials (3–5, 19). Second, when hemorrhagic transformation was evaluated using an ordinal imaging-based framework according to the Heidelberg Bleeding Classification, no significant shift toward more severe hemorrhagic phenotypes was observed between treatment groups (9, 15). Third, baseline stroke severity and onset-to-needle time were independently associated with intracranial hemorrhage (20, 21).

These findings are consistent with randomized trials but our study was not powered to establish comparative safety or non-inferiority. Importantly, unlike prior studies that relied primarily on binary definitions of sICH, our study applied an imaging-centered classification to characterize the full spectrum of hemorrhagic transformation (15, 19). This approach provides greater granularity and reflects the biological heterogeneity of hemorrhagic transformation, which represents a continuum rather than a single clinical entity (9, 13).

Although remote hemorrhagic phenotypes were numerically more frequent in the tenecteplase group, the absolute number of events was small and statistical significance was not reached. Accordingly, these observations should be interpreted with caution. Rather than implying a direct causal effect of tenecteplase, the observed distribution patterns may reflect interactions between systemic fibrinolytic activity and underlying vascular vulnerability in susceptible individuals (13, 14, 22).

Consistent with established literature, higher baseline NIHSS scores and treatment delay were independently associated with hemorrhage (23–25). These results underscore that hemorrhagic risk following thrombolysis appears to be driven primarily by ischemic burden and the timing of reperfusion rather than by the specific thrombolytic agent used (26, 27).

Real-world data remain particularly valuable, as randomized trials often exclude high-risk populations such as elderly patients, individuals with renal dysfunction, or those with large infarct burden—groups known to have an increased risk of hemorrhagic transformation (28–31). Although multivariable analysis did not demonstrate an independent association between thrombolytic agent and sICH, remote hemorrhagic events were observed in several tenecteplase-treated patients (32). Notably, these cases predominantly occurred in individuals with advanced age, renal dysfunction, large-vessel occlusion, fall, elevated, or hyperglycemia (33–36). The selection of thrombolytic agents requires further consideration of these risk factors and necessitates additional research. Given the limited number of events, these findings should be considered exploratory (25, 26).

Several limitations warrant consideration. This was a single-center retrospective study with modest sample size, limiting statistical power for rare hemorrhagic phenotypes such as remote bleeding. The limited number of severe events precluded extensive subgroup analyses. Additionally, infarct volume and perfusion parameters were not available, restricting mechanistic inference.

Future multicenter prospective studies incorporating volumetric and perfusion imaging data are needed to determine whether tenecteplase is associated with distinct spatial hemorrhagic phenotypes in high-risk populations.

## Conclusion

In this exploratory real-world cohort of patients with acute ischemic stroke treated with intravenous thrombolysis, tenecteplase was not associated with an obvious increase in intracranial hemorrhagic events compared with alteplase. Imaging-based assessment using the Heidelberg Bleeding Classification did not reveal a significant shift toward more severe hemorrhagic phenotypes between treatment groups. Higher baseline NIHSS score and longer onset-to-needle time were associated with intracranial hemorrhage, suggesting that baseline stroke severity and treatment delay may contribute more prominently to hemorrhagic risk than thrombolytic agent type in this cohort. However, given the modest sample size and the limited number of severe or remote hemorrhagic events, these findings should be interpreted as preliminary and hypothesis-generating. Larger prospective multicenter studies incorporating detailed infarct volume, vascular occlusion, and perfusion imaging data are needed to further clarify whether tenecteplase is associated with distinct hemorrhagic imaging patterns in high-risk stroke populations.

## Data Availability

The original contributions presented in the study are included in the article/supplementary material, further inquiries can be directed to the corresponding author/s.
